# The Neurorehabilitation of Neurological Movement Disorders Requires Rigorous and Sustained Research

**DOI:** 10.3390/jcm13030852

**Published:** 2024-02-01

**Authors:** Akiyoshi Matsugi, Naoki Yoshida, Hideki Nakano, Yohei Okada

**Affiliations:** 1 Faculty of Rehabilitation, Shijonawate Gakuen University, Osaka 574-0011, Japan; 2 Faculty of Health Sciences, Kansai University of Health Sciences, Osaka 590-0433, Japan; ysd@rehalab.jpn.org; 3 Department of Physical Therapy, Faculty of Health Sciences, Kyoto Tachibana University, Kyoto 607-8175, Japan; nakano-h@tachibana-u.ac.jp; 4 Faculty of Health Science, Kio University, Nara 635-0832, Japan; y.okada@kio.ac.jp

## 1. Introduction

Movement disorders that stem from neurological conditions such as stroke, cerebral palsy, multiple sclerosis (MS), Parkinson’s disease (PD), and spinocerebellar degeneration (SCD) can significantly impair a person’s activities of daily living (ADL). Neurorehabilitation aims to mitigate the impact of these movement disorders, alleviate constraints on the ADL, and promote increased social participation [1]. The advancements in neurorehabilitation necessitate a thorough exploration of the underlying mechanisms of movement disorders, the formulation of pathological hypotheses, and the development of new clinical interventions and assessments, including outcome measure studies. This Special Issue collects contributions from a wide range of domains, including fundamental research that elucidates the intricacies of movement disorders and advancements in evaluation measurement technologies, innovative interventional studies, and prediction of clinical outcome studies.

These studies are expected to augment the recent developments in the field of neurorehabilitation. In this editorial, we review these findings and discuss the future of, as well as the remaining issues in, this field.

## 2. An Overview of the Published Articles

The RALLY Trial, led by Shuji Matsumoto et al., investigated the Walkaide^®^ device’s effectiveness at enhancing the walking ability of post-stroke patients with foot drop (Contribution 1). Despite the lack of significant improvement in the primary outcome measure—the distance covered during a 6-min walk test—this study provides valuable insights into the role of functional electrical stimulation (FES) in Japanese stroke patients during their convalescent phase.

Shin et al. conducted a randomized controlled trial (RCT) to assess the impact of the RAPAEL^®^ Smart Glove digital training system on the upper extremity function and cortical hemodynamic changes in patients with subacute stroke (Contribution 2). This study highlights the potential of game-based virtual reality training to improve motor function and cortical activation, which is consistent with previous studies [2].

Gerber et al. explored hand ownership in teenagers with unilateral cerebral palsy, revealing the difference in their experiences compared to those of typically developing teenagers (Contribution 3). This study emphasizes the importance of understanding the subjective experience of hand ownership and provides insights into early intervention strategies.

Romano et al. (Contribution 4) presented a novel approach that used exergame-based exercise training for children with ataxia. This study demonstrated improvements in their hand dexterity, emphasizing the potential of exergaming to engage children during effective upper-body rehabilitation.

Bedla et al. proposed a novel method for estimating the Gross Motor Function Measure (GMFM) of children with cerebral palsy using the Zebris FDM-T treadmill (Contribution 5). This approach offers a promising alternative for objective diagnostics through its reduction in assessment time and enhanced accessibility.

Mihai et al. investigated the combined effects of radial extracorporeal shock wave therapy and visual feedback balance training on lower limb post-stroke spasticity, trunk performance, and balance (Contribution 6). This study demonstrates the ability of a comprehensive approach to address multiple aspects of post-stroke rehabilitation.

Yang et al. introduced a 3D-printed dynamic hand–wrist splint for chronic stroke patients, and demonstrated how it improved hand dexterity (Contribution 7). This innovative intervention offers a personalized and effective solution for at-home rehabilitation.

Tamaru et al. explored the effects of nerve gliding exercises following carpal tunnel release surgery, emphasizing the importance of the conduction velocity of sensory nerves in predicting hand function improvement (Contribution 8). This study provides valuable insights into postoperative rehabilitation strategies.

In et al. investigated the effects of posterior pelvic tilt taping on the pelvic inclination, muscle strength, and gait ability of stroke patients (Contribution 9). This study highlights the potential benefits of taping as an adjunctive intervention to enhance the postural control and gait of stroke survivors.

A comprehensive review, written by Sîrbu et al., assesses current neurorehabilitation approaches to multiple sclerosis, emphasizing the need for personalized strategies to address the varied symptoms of this prevalent neurological disorder (Contribution 10).

Marcos-Antón et al. conducted a systematic review of the use of the MYO Armband^®^ for assessing and training manual dexterity in individuals with upper limb impairment (Contribution 11). Their work provides a comprehensive overview of the accuracy and clinical effects of this technology and offers valuable guidance for practitioners.

Rodríguez-Fuentes et al. systematically reviewed the therapeutic effects of the Pilates method for patients with multiple sclerosis (Contribution 12). While highlighting positive outcomes in terms of balance, gait, and physical functional conditions, this review emphasizes the need for larger-scale studies with well-defined protocols.

## 3. Knowledge Gaps and Further Study

In summation, the impairments addressed by these neurological disease studies are gait disturbance, balance impairment, and upper extremity utility. These are in line with the unmet rehabilitation needs most sought after by neurological disorder patients. The studies published in this Special Issue have made valuable contributions to the field of neurorehabilitation; however, they also reveal persistent knowledge gaps that require further exploration. Key areas for future investigation include more robust large-scale clinical trials that encompass diverse participant populations. Additionally, long-term follow-up studies are necessary to evaluate the sustained effects of these interventions. Comparative effectiveness research is needed to identify the most efficacious rehabilitation strategies, while the exploration and integration of innovative technologies into neurorehabilitation practices are promising for generating advancements in the field. Furthermore, a comprehensive understanding of individualized rehabilitation plans based on patient-specific factors is essential for tailoring interventions to meet diverse patient needs and optimize their outcomes. For example, in this SI, there was no positive effect found in the functional electrical stimulation of ankle muscle on gait speed, despite a well-controlled study (Contribution 1). However, this intervention study should be conducted other view such as other outcome, other population, and other stimulation conditions. 

Selecting optimal outcomes is crucial to advancing clinical reasoning, just as clinical research is to selecting optimal interventions. In this SI, upper limb functions were measured in a number of important studies, in patients with cerebral palsy, peripheral nerve injury, and post stroke (Contributions 2, 3, 4, 7, 8, and 11). However, there is no internationally agreed-upon outcome to be obtained for these diseases, which hinders the future consolidation of these findings. Core outcome sets (COSs) are necessary to advance clinical research [3]; however, their development is lacking for certain conditions, even for common incurable neurological diseases, such as SCD and PD, which are targets of neurorehabilitation. We must also initiate the development of COSs for the treatment of neurological diseases. 

While the narrative and literature reviews reported in this SI (Contribution 7) are important in establishing the efficacy of neurorehabilitation treatments, more systematic reviews should also to be conducted to proactively examine the effectiveness of rehabilitation interventions in conditions such as stroke [4], PD [5], and SCD [6]. Furthermore, guidelines [7] must be developed so that physicians, physical therapists, occupational therapists, and patients worldwide can choose the evidence-based treatment that will lead to the best outcome. Additionally, clinicians must be educated on how to utilize the findings of systematic reviews. 

Neuromodulation technology plays an important role in promoting neurorehabilitation [8]. Transcranial electrical stimulation and repetitive transcranial magnetic stimulation are now being validated for stroke [9], SCD [10], and PD [11]. However, there are few RCTs of this technology, and parameter selection methods for the population conditions and individual cases that are expected to find this technology effective remain unclear. In addition to conventional physical and occupational therapies, we must continue to examine the efficacy of neuromodulation.

Neuroregenerative medicine [12] for spinal cord injuries [13], stroke, and neurodegenerative diseases, such as PD and SCD, is expected in the near future. Neurorehabilitation after structural regeneration may be effective in restoring function to patients, and this issue should be addressed in the future.

## 4. Conclusions

The field of neurorehabilitation continues to evolve, with innovative interventions being accompanied by outcome studies aimed at addressing the complex challenges posed by various neurological conditions. While advancements in wearable technology, such as virtual reality and noninvasive brain stimulation, offer promising avenues for rehabilitation, the multifaceted nature of neurological disorders requires continuous research and collaboration to optimize treatment strategies. This editorial underscores the progress that has been made and highlights the avenues for future exploration within the dynamic landscape of neurorehabilitation.
